# Out‐of‐Pocket Costs and Financial Toxicity Associated With the Surgical Management of Oesophageal Cancer

**DOI:** 10.1111/ans.70514

**Published:** 2026-02-05

**Authors:** Josipa Petric, Muktar Ahmed, Chris Trethewey, John Clements, Tim Bright, David I. Watson, Norma B. Bulamu

**Affiliations:** ^1^ Flinders Health and Medical Research Institute, College of Medicine and Public Health Flinders University Adelaide South Australia Australia; ^2^ Flinders Health and Medical Research Institute, Consumer Involvement Steering Committee, College of Medicine and Public Health Flinders University Adelaide South Australia Australia; ^3^ Consumer Advisory Board for the Cancer Council Adelaide UK; ^4^ Department of Surgery Flinders Medical Centre Adelaide South Australia Australia

**Keywords:** cancer survivorship, financial toxicity, oesophageal cancer, out‐of‐pocket costs

## Abstract

**Background:**

Patients undergoing cancer treatment incur significant out‐of‐pocket costs attributed to both medical and non‐medical expenditure. We quantified out‐of‐pocket costs for patients receiving surgical treatment for oesophageal cancer and their financial toxicity.

**Methods:**

Patients who had undergone oesophagectomy for cancer completed an out‐of‐pocket questionnaire which determined medical costs (e.g., gap payments and medications), non‐medical costs (e.g., travel, accommodation, wage loss) and carer costs (travel and wage loss). Financial toxicity was assessed using the validated Comprehensive Score for Financial Toxicity (COST) questionnaire. Out‐of‐pocket costs and financial toxicity were summarised using medians with bootstrapped 95% confidence intervals (CIs) (1000 resamples). Between‐group comparisons were assessed with Wilcoxon rank‐sum and Kruskal–Wallis tests and associations with income percentiles using Spearman's correlation.

**Results:**

Seventy individuals completed the survey (43.3% response rate). The majority were male (85.7%), aged 60–79 (76.5%) and 0–5 years post‐cancer diagnosis (55.7%). Median out‐of‐pocket expenditure was $1352 and was mainly attributed to wage loss (64.7%), followed by carer cost (23.7%). Out‐of‐pocket costs were higher for younger age groups (40–59 years) compared to those aged 60–79 years (*p* = 0.003). There was no statistically significant difference in out‐of‐pocket costs between public versus privately insured patients. Median out‐of‐pocket costs trended higher for rural ($1696) versus urban located patients ($1235), but this was not statistically significantly different (*p* = 0.140). The median financial toxicity score was 23.5 (95% CI: 21.0–27.5), indicating moderate financial toxicity. Financial toxicity did not differ significantly by age, gender, country of birth, education or location. A lower income percentile was associated with greater financial toxicity (*ρ* = −0.30, *p* = 0.012).

**Conclusion:**

Patients facing oesophagectomy for cancer incur many out‐of‐pocket costs, mostly due to wage loss from time spent away from work for both patients and carers. Younger patients and those with lower income face proportionately greater financial burdens, highlighting a need for targeted support to reduce financial stress.

## 1 Introduction

Cancer and its treatment impose a cost burden on patients and their families. The cost of treatment depends on multiple factors including the type and location of cancer, cancer stage, treatment setting and whether patients have private health insurance or not [1]. Even in Australia where healthcare resources are abundant, 15%–32% of the treatment cost of cancer can be funded by patients and their families as a copayment [2]. Patients from remote locations also incur non‐medical costs such as travel and accommodation [2].

Estimates from a study of patients undergoing cancer care from 2006 to 2014 reported direct health system mean annual costs after initial treatment of $4474/case and a 10‐fold increase in this cost in the last year of life [1]. In a 2020 study from New South Wales, additional out‐of‐pocket costs of more than $1000/year for health care were reported from a survey of 45 061 cancer patients [3]. Furthermore, a systematic review found out‐of‐pocket costs ranged from $977 to $11 077 in Australia depending on the type of cancer and any treatment complications [4]. In a more recent study from Queensland evaluating head and neck cancers, out‐of‐pocket costs ranged from a median of $1796 to $25 050, and higher financial toxicity scores were associated with poorer health related quality of life outcomes [5].

Significant out‐of‐pocket expenditures might also affect how patients access and comply with treatment, with patients in financial difficulty missing appointments, delaying or foregoing treatment or choosing an alternative treatment and this can impact survival [2]. It is proposed that this is more likely for rural and remotely located patients, who account for 28% of the Australian population [6]. This group has previously been found to incur higher out‐of‐pocket costs [2]. In addition, patients of lower socioeconomic status are more likely to be diagnosed with cancer at a later stage and are more susceptible to financial distress [7].

For patients diagnosed with oesophageal cancer these issues can be, particularly, problematic. The workup requires extensive investigation with scans and fitness assessments, which are often only available in urban locations. Potentially curative treatment entails chemotherapy and sometimes concurrent radiotherapy before oesophagectomy. Oesophagectomy can only be performed in appropriately resourced centres, usually requires a hospital stay of at least 1–2 weeks, and it can be followed by significant complications which then extend the hospital stay and recovery period. For these reasons, oesophageal cancer might place a significantly higher financial burden on patients and families, and for rural and remotely located patients this could be even more problematic. While hospital treatment costs have been quantified for oesophageal cancer [8, 9], out‐of‐pocket costs in Australia are poorly understood. Hence, the aim of this study was to quantify the out‐of‐pocket costs incurred by patients receiving surgical treatment for oesophageal cancer, and to determine if factors such as age and metropolitan versus rural/remote location impact these costs.

## 2 Methods

Patients who had previously undergone an oesophagectomy for oesophageal cancer (adenocarcinoma or squamous cell carcinoma) between 2000 and 2022 at Flinders Medical Centre, with at least 24 months follow‐up after surgery were considered for this study. Participants were excluded if they could not read and understand English or were known to be deceased. Between January and August 2024, potential participants were mailed a questionnaire which addressed out‐of‐pocket costs and financial toxicity. If the questionnaire was not returned, a reminder phone call was made 2 weeks later to encourage completion.

The aim of the study was to assess the financial burden associated with treatment for oesophageal cancer experienced by patients and their carers. The primary outcome of interest was out‐of‐pocket costs. For patients, this was defined as medical or other expenses incurred that were not reimbursed by health insurers, Medicare or other funders; non‐medical costs, which included travel and accommodation costs; and loss of wages. In addition, carer costs were also determined and these included additional travel expenses and loss of wages. Medical out‐of‐pocket costs included gap fee payments for consultations, investigations, treatments, medications and other healthcare services related to care of the oesophageal cancer.

The secondary outcome for this study was the financial toxicity score, which was calculated using the COST questionnaire [10]. The COST questionnaire is a validated measure of the financial distress experienced by patients [10]. Financial toxicity scores range from 1 to 44, with lower scores indicating greater financial distress. Financial toxicity scores were compared across sociodemographic groups using non‐parametric tests.

Other information collected included sociodemographic characteristics (age, sex, marital status, education level, country of birth and Australian residential postcode) and self‐reported income‐related factors (e.g., household income, percentage of income spent on cancer care). Additional information determined treatment‐related costs including type of visit, presence of a support person during appointments, time off work for appointments, gross income, date of diagnosis, insurance type (private vs. public) and time since diagnosis to completion of the questionnaire. The full questionnaire is provided in Appendix A in the Supporting Information.

Sociodemographic and clinical characteristics were summarised descriptively. Costs were categorised and summed as follows:

- *Total medical costs*: included consultation and investigation gap fees and medications.
- *Total non‐medical costs*: included travel expenses, accommodation and self‐reported wage and income loss.
- *Carer costs*: included travel, accommodation and wage loss of accompanying carers.
- *Total out‐of‐pocket costs*: Sum of all patient and carer costs.

All costs were expressed in Australian dollars. Data are reported as median and 95% confidence intervals (CIs). Analysis examined the distribution of out‐of‐pocket costs and financial toxicity scores, alongside sociodemographic characteristics, to assess the financial burden on oesophageal cancer survivors and their carers. For out‐of‐pocket costs, comparisons were made across different sociodemographic groups. As the Shapiro–Wilk test indicated non‐normality (*p* < 0.05), non‐parametric methods were used. The Wilcoxon rank‐sum test was applied to compare costs between urban and rural participants, between age groups (40–59 vs. 60–79) and between insurance types (private vs. public). Levene's test confirmed equal variances for age group comparisons (*p* = 0.405), but parametric tests were not used due to the violation of normality assumptions. Median costs and 95% CIs were estimated using bootstrap resampling (1000 iterations).

Financial toxicity scores showed no evidence of deviation from normality (Shapiro–Wilk *p* = 0.93). However, for consistency with the out‐of‐pocket cost reporting and because financial toxicity scores were markedly skewed in distributional shape, they were summarised using the median and a bootstrapped 95% CI (1000 resamples). Group differences in financial toxicity scores were assessed using non‐parametric tests, including the Wilcoxon rank‐sum test for binary comparisons (age groups, genders, residential area, insurance types (private vs. public) and country of birth) and the Kruskal–Wallis test for multi‐category variables (education levels, marital status and grouped time gap of diagnosis). The association between income percentile and financial toxicity was evaluated using Spearman's rank correlation.

All statistical analyses were conducted using R version 4.3.2 for Windows (R Core Team, R Foundation for Statistical Computing, Vienna, Austria) or SPSS Statistics version 28 for MacBook (IBM SPSS Statistics for Macintosh, Version 28, Armonk, TY, USA), with significance set at two‐sided *p* < 0.05. Ethical approval was obtained from the Southern Adelaide Clinical Research Ethics Committee and informed consent was obtained from all study participants (Reference No. 187.23—LNR/23/SAC/187).

## 3 Results

### 3.1 Participant Characteristics

Questionnaires were sent to 178 individuals and 77 were returned (43% response rate). Complete data was available from 70 responses as not all questionnaires were fully completed. Respondent characteristics are summarised in Table 1. Most respondents were aged between 60 and 79 years (76.5%), male (85.7%), married or in a de facto relationship (79.7%), born in Australia (76%) and lived in urban areas (63%). Education levels were varied; 38% had completed university or higher education, 29% had completed secondary school and 21% had technical or vocational training.

**TABLE 1:** Bivariate analysis of out‐of‐pocket costs and financial toxicity scores by participant characteristics (*N* = 70).

Characteristic	*N* (%)	Out‐of‐pocket cost		Financial toxicity score	
		Median (95% CI) AUD	*p*	Median (95% CI) AUD	*p*
Gender			0.046		0.677
Male	60 (85.7)	$1476 (1103–2912)		$23 (21–28)	
Female	10 (14.3)	$302 (80–2136)		$28 (19–31)	
Age group (years)			0.003		0.065
40–59	16 (23.5)	$13 062 (2213–28 417)		$22 (17–25)	
60–79	52 (76.5)	$1060 (636–1696)		$26 (21–30)	
Insurance type			0.314		0.917
Private	20 (26.5)	$1798 (1126–4852)		$25 (17–31)	
Public	50 (73.5)	$1192 (658–2178)		$23 (21–28)	
Education level			0.475		0.317
Primary school	3 (1.4)	$212 (212–212)		$30 (30–30)	
Secondary	21 (30.0)	$2136 (1040–5493)		$22 (17–27)	
Trade/diploma	27 (38.6)	$1696 (608–13 832)		$23 (20–30)	
Bachelor's	15 (21.4)	$1235 (434–2085)		$28 (24–32)	
Postgraduate	4 (5.7)	$1393 (35–4212)		$23 (7–35)	
Marital status			0.454		0.094
Married	56 (80.0)	$1353 (986–2327)		$25 (22–30)	
Single	3 (4.3)	$2136 (317–19 001)		$23 (10–31)	
Widowed	1 (1.4)	$35 (35–35)		$36 (36–36)	
Divorced/separated	10 (12.9)	$2220 (63–5493)		$19 (10–28)	
Born in Australia			0.724		0.962
Yes	53 (75.7)	$1400 (986–2327)		$25 (21–28)	
No	17 (22.9)	$1149 (642–3775)		$22 (20–31)	
Postcode classification			0.144		0.074
Urban	44 (62.9)	$1235 (608–2165)		$26 (23–30)	
Rural	26 (37.1)	$1696 (1057–5953)		$20 (17–26)	
Time since diagnosis			0.125		0.398
0–5 years	39 (55.7)	$1235 (753–2136)		$25 (22–31)	
5–10 years	23 (32.9)	$1360 (470–3775)		$24 (17–27)	
10–15 years	5 (5.7)	$879 (35–20 233)		$18 (10–28)	
15–20 years	3 (4.3)	$25 995 (12 292–105 776)		$20 (17–26)	
Income percentile			0.322		0.141
0	43 (67.2)	$753 (455–3300)		$27 (22–30)	
1	13 (20.3)	$1512 (1235–2490)		$23 (18–26)	
2	7 (10.9)	$2164 (1040–20 233)		$17 (12–28)	
3	1 (1.6)	$470 (470–470)		$12 (12–12)	

*Note: p*‐values are from non‐parametric tests (*Wilcoxon rank‐sum test*) due to the non‐normal distribution of out‐of‐pocket cost variables, presented as medians with 95% confidence intervals estimated using bootstrap resampling (1000 iterations).

Abbreviation: CI = confidence interval.

### 3.2 Out‐of‐Pocket Costs

**FIGURE 1:**
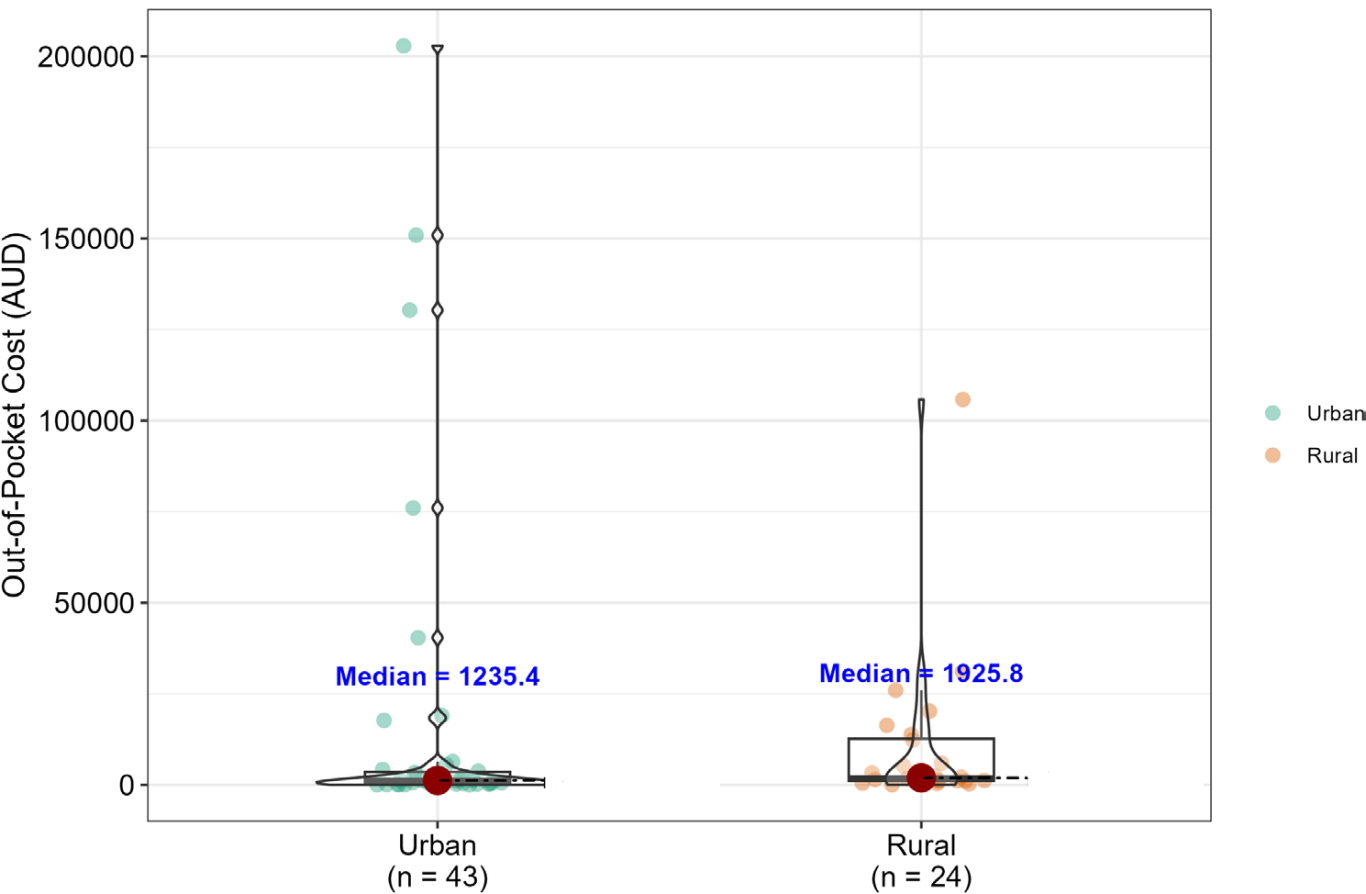
Comparison of out‐of‐pocket costs between urban versus rural participants. This figure shows the distribution of patient out‐of‐pocket costs (Australian Dollar) by area of residence. Rural participants had a higher median out‐of‐pocket cost ($1925) compared with urban participants ($1235), but this difference was not statistically significant (Wilcoxon rank‐sum test, *p* = 0.14). Medians are annotated above each box.

The median out‐of‐pocket cost was $1352 (range: $17–$202 854; interquartile range: $4839; standard deviation $36 646). Mean out‐of‐pocket costs were substantially higher at $13 885, reflecting a strong right‐skewed distribution driven by a small number of patients reporting exceptionally high expenses. Urban patients had median out‐of‐pocket costs of $1235 (95% CI: $608–$2164) compared with $1696 (95% CI: $1057–$5953) for rural patients (Table 1). This difference was not statistically significant (Wilcoxon rank sum test, *p* = 0.14) (Figure 1).

**FIGURE 2:**
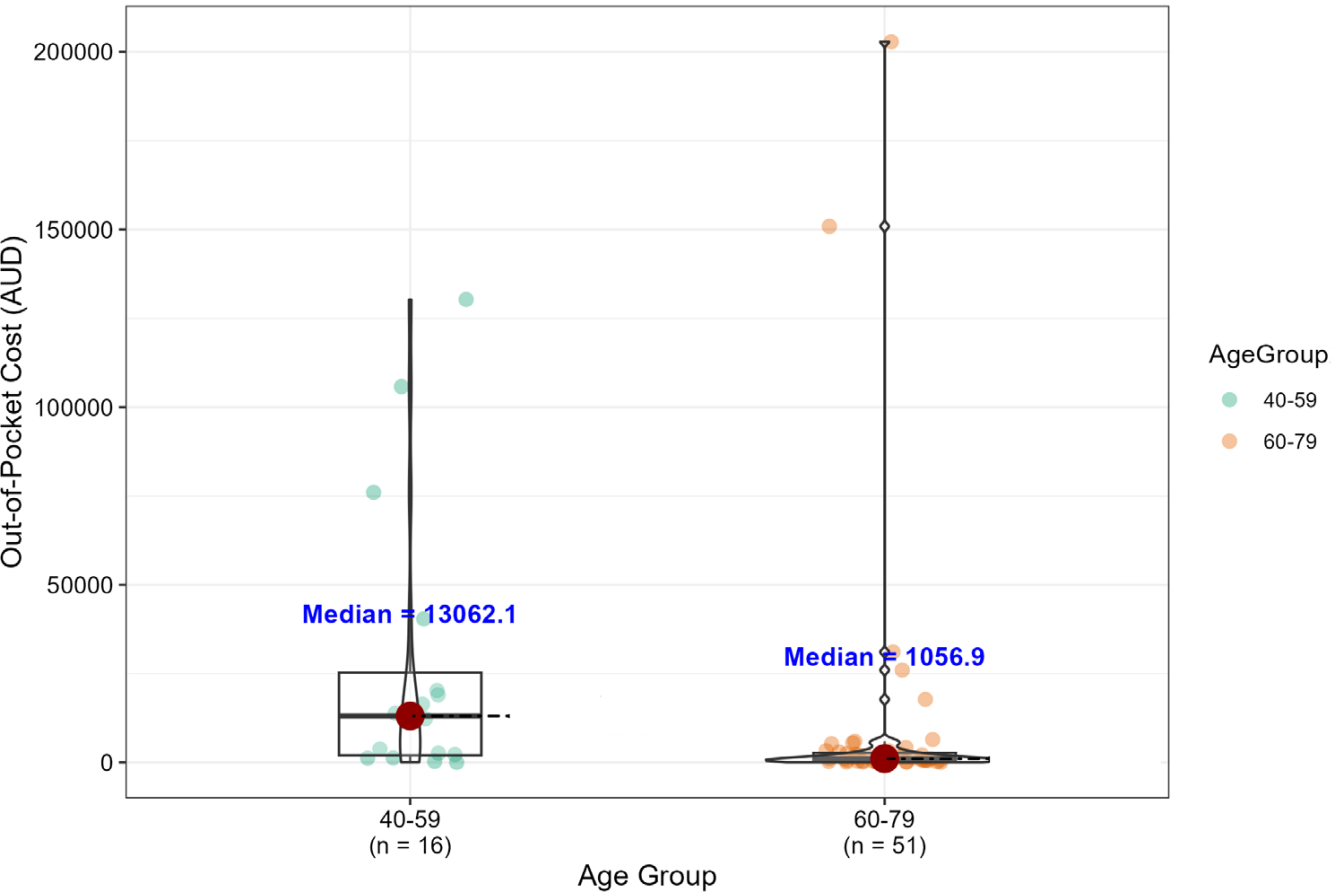
Comparison of out‐of‐pocket costs across age groups 40–59 and above 60. Displayed are the distributions of out‐of‐pocket costs (Australian Dollar) among participants aged 40–59 and 60–79 years. The younger age group incurred a markedly higher median cost ($13 062) than the older group ($1057), reflecting the substantial wage‐loss component among working‐age patients. The difference between groups was statistically significant (Wilcoxon rank‐sum test, *p* = 0.003). Medians are annotated above each box.

Out‐of‐pocket costs differed significantly between age groups (40–59 vs. 60–79 years) with the younger patients incurring significantly more costs (median $13 062; 95% CI: $2220–$20 232 vs. $1057; 95% CI: $636–$1696; *p* = 0.003) (Table 1, Figure 2). The proportion of younger versus older patients was similar within each postcode classification (40–59 years: 23% urban vs. 24% rural). The median costs for public patients were $1192 (95% CIs: $658–$2192) vs. $1798 (95% CIs: $1126–$4852) for privately insured patients. This difference was not statistically significant (*W* = 377, *p* = 0.31). A detailed itemization of out‐of‐pocket cost components by insurance type is presented in Table S1, showing that medical costs differed modestly between private versus public patients.

Based on median component costs, medical expenses represented the largest individual out‐of‐pocket cost across participants (median $514; 95% CI: $305–$880), followed by travel costs (median $67; 95% CI: $35–$122), while accommodation, wage loss and carer costs had median values of $0 (Table 2). When disaggregated by age group, participants aged 40–59 years had substantially higher median medical costs ($1392; 95% CI: $280–$2193 vs. $420; 95% CI: $291–$835). Travel‐related expenses were similar between the two groups, with overlapping CIs ($69 in 40–59 years vs. $70 in 60–79 years). Median values for accommodation, wage loss and carer costs were $0 in both age groups due to zero‐inflation in these components. A similar pattern was observed when stratified by urban and rural residence. Urban participants had higher median medical costs ($622; 95% CI: $331–$1192) compared with rural participants ($388; 95% CI: $220–$905), whereas rural participants had higher median travel costs ($273; 95% CI: $9–$496 vs. $55; 95% CI: $25–$118). Median values for accommodation, wage loss and carer‐related expenses were $0 in both groups (Table 2).

**TABLE 2:** Total and median out‐of‐pocket costs by component for all participants, and separately for urban and rural residents.

Components	All participants			Urban			Rural		
	Total cost all (AUD)	%	Median (95% CI)	Total cost (AUD)	%	Median (95% CI)	Total cost (AUD)	%	Median (95% CI)
Medical	$74 625	7.9	514 (305–880)	$51 516	7.5	622 (331–1192)	$23 108	9.0	388 (220–905)
Travel	$30 813	3.3	67 (35–122)	$5720	0.8	55 (25–118)	$25 093	9.8	273 (9–496)
Accommodation	$4100	0.4	0 (0–0)	$0.00	0.0	0 (0–0)	$4100	1.6	0 (0–0)
Wage loss	$611 056	64.7	0 (0–0)	$437 612	63.6	0 (0–0)	$173 444	67.6	0 (0–0)
Carer cost	$223 598.	23.7	0 (0–0)	$192 848	28.0	0 (0–0)	$30 750	12.0	0 (0–0)

*Note*: Total costs represent the aggregated expenditure across the cohort, whereas median values reflect the typical (per‐patient) cost within each component. Many components show a median of zero due to large proportions of participants without expenses in those categories.

Abbreviation: CI = confidence interval.

### 3.3 Financial Toxicity

The median financial toxicity score was 23.50 (95% CI: 21.0–27.5). Respondents from urban areas had a higher median financial toxicity score (26.0, 95% CI: 23–30) than those from rural areas (20.0, 95% CI: 17–26), but this difference was not statistically significant (*p* = 0.074). Similarly, there was no significant difference between aged groups (40–59 years median = 22.0; 95% CI: 17–25, 60–79 years: median = 26.0; 95% CI: 21–30, *p* = 0.115). No significant difference in financial toxicity scores was observed between public vs. private insurance holders (median = 23.0; 95% CI: 21–28 vs. 25.00; 95% CI: 17–31, *p* = 0.962) (Table 1). Financial toxicity scores did not significantly differ by gender, country of birth, age group or education level. However, the income percentile, a proxy of the socioeconomic status, was negatively correlated with financial toxicity (*ρ* = −0.30, *p* = 0.012), indicating that individuals with higher incomes reported lower levels of financial distress (Table 1).

## 4 Discussion

We evaluated the out‐of‐pocket costs for patients with oesophageal cancer treated in South Australia. The median out‐of‐pocket cost was $1352 but ranged widely with some patients spending as much as $202 854 and others as little as $17, highlighting significant variation. Most of the out‐of‐pocket costs were attributed to wage loss for both patients and carers. There was a trend of urban patients incurring higher costs than rural patients, although this difference did not reach statistical significance (*p* = 0.14). However, the distribution of the out‐of‐pocket costs was highly right‐skewed, driven by a small number of urban patients with exceptionally high wage‐loss values. This pattern reflects known higher average incomes for individuals living in urban areas compared to rurally located individuals. As expected, rural patients did report increased costs from travel and accommodation expenses. Although these costs were higher, they remained smaller in magnitude than the wage‐loss burden observed among urban patients, possibly explaining why rural patients had higher median costs despite greater cost variability in the urban group. Consistent with loss of income driving out‐of‐pocket costs, younger patients also reported significantly higher costs (*p* = 0.003). Despite these differences, the overall pattern suggests that rural patients are more likely to incur significant out‐of‐pocket expenses, particularly due to travel‐related costs.

The observed four‐point higher financial toxicity score in urban versus rural participants in our study, even though not statistically significant, aligns with higher mean out‐of‐pocket costs for urban patients, suggesting that higher expenses might contribute to greater financial distress. However, financial toxicity was negatively correlated with higher income percentiles, as individuals with higher incomes reported lower financial distress, even though their out‐of‐pocket expenses were likely greater.

Our findings are consistent with a 2021 systematic literature review of studies from 12 countries, including Australia, which concluded that out‐of‐pocket costs for cancers‐in‐general were comparatively higher in low/middle income countries in populations living in rural settings as well as in low‐income patients and caregivers [11]. It is not surprising that rural patients incur higher costs as it is known that many costs are related to accommodation and travel to an urban treatment site. Having said this, in a 2020 study from Western Australia, rural patients did not have higher out‐of‐pocket costs but instead outer metropolitan residents had to pay more [12]. In Australia, State Health Department schemes that assist rurally located patients with health related travel expenses generally only subsidise a portion of the accommodation and travel, and the difference between cost and reimbursement can be significant for some patients [13]. However, in our study's setting, although rural patients had somewhat higher median costs, the actual difference was not statistically significant. Patients travelling to Adelaide for treatment receive a subsidy from the South Australian government which is taken as full payment for accommodation in Adelaide when staying at Cancer Council SA accommodation. This arrangement ensures patients are not out of pocket for accommodation costs in SA, a consideration that might account for this outcome in our study.

A 2022 systematic review of 18 studies across 10 countries, including Australia, China, the United Kingdom and the United States, found that 14 studies (77.8%) reported significant patient and caregiver loss of income and productivity [14]. In particular, self‐employed patients had limited access to government support and accounted for higher losses, in addition to a lower level of education, being of an ethnic minority, receiving chemotherapy and having an advanced stage of diagnosis [14]. This is consistent with earlier studies which concluded that patients with lower education levels and advanced cancer stages are more likely to experience loss of income [15, 16]. Similarly, in a 2021 systematic review of out‐of‐pocket costs and financial burdens of Australian patients from 19 studies, it was found that younger patients (< 65 years of age) had significantly worse financial burden [4].

In Australia, Medicare ensures all patients can access care in public hospitals free of charge, and there are also incentives to encourage Australians to take out private health insurance and access private hospitals. However, care in private hospitals can result in gap fees which then result in out‐of‐pocket costs [17]. A 2020 study from Western Australia found that patients with cancer who had private health insurance and undergoing surgery had higher out‐of‐pocket expenses [12].

Financial toxicity has been shown to be associated with baseline demographics such as rural and remote location, socioeconomic status and unemployment [18, 19]. Younger patients, with fewer savings, are also at highest risk of bankruptcy [18, 19]. Higher financial toxicity, which is experienced by patients with lower income, is also associated with higher psychological distress and lower quality of life [19]. While the Australian Government does assist with costs by offering services such as a pension, carer payment or disability support, these may be difficult to access or not offer enough to support many of the patients our study identified as experiencing financial toxicity. The quality of care and healthcare equity in these patients can be impacted as they potentially cannot pay the out‐of‐pocket costs incurred with delivery of recommended treatments [3].

There are several limitations in our study, including a relatively small sample size from a single centre which may have introduced selection bias. However, Flinders Medical Centre has a large catchment area which extends all the way to Darwin in the Northern Territory, as well as Western New South Wales and Victoria. This allowed wide capture of socioeconomic statuses across the study, with a significant proportion of the cohort being pensioners, and also a significant proportion reporting a high income. It is possible that the response rate could have been influenced by participant experience, with those who experienced higher financial burden more motivated to respond or alternatively not able to respond as they potentially felt that they were let down or did not have the time to respond due to significant financial stressors. We do not know if responder bias was present or impacted the study outcome, although our results are similar to other studies evaluating other cancers.

Another limitation is the inclusion of patients treated over a 20‐year period. As out‐of‐pocket costs were not adjusted for inflation, this might affect responses from different follow‐up time points and then lead to either underestimation or overestimation of the financial burden for earlier versus later patients. Further, we only focused on patients who had received an oesophagectomy as part of their treatment for oesophageal cancer. Patients undergoing non‐surgical treatments or palliation are also likely to experience significant out‐of‐pocket costs, and our study has not assessed those scenarios. Finally, while we identified wage loss, we did not ask any direct questions about job loss or the possibility of some patients retiring earlier than intended, and the potential long‐term financial impacts of these events if they occurred. We also did not stratify for employment type or assess access to income protection insurance or associated schemes. However, as these offset costs they were implied in the reported time off work and associated wage loss.

In conclusion, patients undergoing oesophagectomy for oesophageal cancer can face substantial financial burden during and after treatment. The major contributor to out‐of‐pocket expenditure is wage loss for the patient and their carer, and this can result in significant financial distress and potentially affect treatment adherence, follow‐up engagement and overall quality of life. These findings provide the detailed cost data in the setting of oesophageal cancer surgery in Australia and can be used to inform health economic models and guide future service design. Contrary to expectations, rural patients did not report higher costs, suggesting that supports for travel and accommodation are mitigating geographic inequities. However, the dominant impact of lost income among younger patients and higher income individuals highlights a need for policies supporting income protection, employment reintegration and targeted financial counselling for working‐age cancer survivors.

## Author Contributions

**Tim Bright:** conceptualization, investigation, funding acquisition, writing – original draft, validation, writing – review and editing, visualization, supervision, resources. **David I. Watson:** resources, supervision, data curation, project administration, writing – review and editing, visualization, validation, methodology, conceptualization, investigation, funding acquisition, writing – original draft. **Josipa Petric:** conceptualization, investigation, funding acquisition, writing – original draft, methodology, validation, visualization, writing – review and editing, project administration, data curation, resources, formal analysis. **Norma B. Bulamu:** conceptualization, investigation, funding acquisition, writing – original draft, methodology, validation, visualization, writing – review and editing, formal analysis, software, project administration, data curation, supervision, resources.

## Funding

Josipa Petric was supported by a National Health and Medical Research Council Postgraduate Scholarship, Muktar Ahmed was supported by a Cancer Council South Australia Beat Cancer grant and Norma Bulamu was supported by a Cancer Council South Australia Beat Cancer Early Career Researcher fellowship.

## Conflicts of Interest

The authors declare no conflicts of interest.

## Supporting information


**Appendix A.** A copy of the patient questionnaire.
**Table S1:** The breakdown of total out‐of‐pocket costs based on the components across all participants, and by insurance type.
**Supplementary File 1**. Participant questionnaire.
**Table S1:** Total out‐of‐pocket costs based on the components across all participants, and by insurance type.

## Data Availability

The data that support the findings of this study are available on request from the corresponding author. The data are not publicly available due to privacy or ethical restrictions.
