# Supplementary material for: Out‐of‐Pocket Costs and Financial Toxicity Associated With the Surgical Management of Oesophageal Cancer
Source: ANZ J Surg. 2026 Feb 5;96(4):881–8. doi: 10.1111/ans.70514 (PMC13129250; doi:10.1111/ans.70514)
Supplement: Supplementary file 1 — Appendix A. A copy of the patient questionnaire. Table S1: The breakdown of total out‐of‐pocket costs based on the components across all participants, and by insurance type. Supplementary File 1. Participant questionnaire. Table S1: Total out‐of‐pocket costs based on the components across all participants, and by insurance type. [file ANS-96-881-s001.docx]

**SUPPLEMENTARY FILE**

**Appendix A** – a copy of the patient questionnaire.

**INDIRECT OR OUT-OF-POCKET COSTS ASSOCIATED WITH OESOPHAGEAL CANCER SERVICES**

**(Questionnaire to be returned in the attached envelope)**

**SECTION A: COST**

Below is a list of statements that other people with oesophageal cancer have said are important. **Please circle or mark one number per line to indicate your response as it applies to the past 7 days.**

| **STATEMENT** | Not at all | A little bit | Somewhat | Quite a bit | Very much | N/A |
| --- | --- | --- | --- | --- | --- | --- |
| **A1.** I know that I have enough money in savings, retirement, or assets to cover the costs of my treatment. | 0 | 1 | 2 | 3 | 4 | N/A |
| **A2.** My out-of-pocket medical expenses are more than I thought they would be. | 0 | 1 | 2 | 3 | 4 | N/A |
| **A3.** I worry about the financial problems I will have in the future as a result of my illness or treatment. | 0 | 1 | 2 | 3 | 4 | N/A |
| **A4.** I feel I have no choice about the amount of money I spend on care. | 0 | 1 | 2 | 3 | 4 | N/A |
| **A5.** I am frustrated that I cannot work or contribute as much as I usually do. | 0 | 1 | 2 | 3 | 4 | N/A |
| **A6.** I am satisfied with my current financial situation. | 0 | 1 | 2 | 3 | 4 | N/A |
| **A7.** I am able to meet my monthly expenses | 0 | 1 | 2 | 3 | 4 | N/A |
| **A8.** I feel financially stressed. | 0 | 1 | 2 | 3 | 4 | N/A |
| **A9.** I am concerned about keeping my job and income, including paid work at home. | 0 | 1 | 2 | 3 | 4 | N/A |
| **A10.** My cancer or treatment has reduced my satisfaction with my present financial situation. | 0 | 1 | 2 | 3 | 4 | N/A |
| **A11.** I feel in control of my financial situation. | 0 | 1 | 2 | 3 | 4 | N/A |
| **A12.** My illness has been a financial hardship to my family and me. | 0 | 1 | 2 | 3 | 4 | N/A |

**SECTION B: ADDITIONAL COSTS**

**B1. From the time of your admission for your surgery until now, how many times have you visited the hospital, your GP, physiotherapist, speech therapist, dietician or other medical professional regarding your diagnosis of oesophageal cancer?** *If you are unable to recall the exact number of visits, please give your best estimate.*

☐ Only attended for the surgery – not re-attended since or seen the GP

☐ Multiple visits:

| **Type of Visit** | **Private car** | **Taxi** | **Number of visits** |
| --- | --- | --- | --- |
| Hospital | ☐ | ☐ |  |
| Your GP | ☐ | ☐ |  |
| Physiotherapist | ☐ | ☐ |  |
| Dietician | ☐ | ☐ |  |
| Speech therapist | ☐ | ☐ |  |
| **Other medical professional -** Please specify which type | | | |
|  | ☐ | ☐ |  |
|  | ☐ | ☐ |  |
|  | ☐ | ☐ |  |

**B2. How much did it cost you in total for travel for each visit?** Please include the fare each time for the taxi, or car mileage from home to hospital and cost of parking. *Please do not include any out-of-pocket gap payments. If you are unable to recall the exact cost, please give your best estimate.*

| **Type of Visit** | **Cost ($)**  *(Only parking fees if private car)* | **Mileage (km)**  *Leave blank if taxi* |
| --- | --- | --- |
| Hospital |  |  |
| Your GP |  |  |
| Physiotherapist |  |  |
| Dietician |  |  |
| Speech therapist |  |  |
| **Other medical professional -** Please specify which type | | |
|  |  |  |
|  |  |  |
|  |  |  |

**B3. Were there any out-of-pocket gap payments for these visits and if so, how much did they cost?**

| **Type of Visit** | **Yes** | **No** | **N/A** | **Cost ($)** |
| --- | --- | --- | --- | --- |
| Hospital | ☐ | ☐ | ☐ |  |
| Your GP | ☐ | ☐ | ☐ |  |
| Physiotherapist | ☐ | ☐ | ☐ |  |
| Dietician | ☐ | ☐ | ☐ |  |
| Speech therapist | ☐ | ☐ | ☐ |  |
| **Other medical professional -** Please specify which type | | | | |
|  | ☐ | ☐ | ☐ |  |
|  | ☐ | ☐ | ☐ |  |
|  | ☐ | ☐ | ☐ |  |

**B4. How often were you accompanied by a partner or close friend or support person for your visits?**

☐ None of the time ☐ 1-5 times ☐ 5-10 times ☐ 10-15 times

☐ Every time

**B5. Did the person accompanying you travel with you in the same vehicle?**

☐ Yes ☐ No ☐ N/A

1. **If not, how much did they pay for their travel (not including accommodation)?** *If you are unable to recall the exact cost, please give your best estimate.*

| **Type of Visit** | **Cost ($)**  *(Only parking fees if private car)* | **Mileage (km)**  *Leave blank if taxi* |
| --- | --- | --- |
| Hospital |  |  |
| Your GP |  |  |
| Physiotherapist |  |  |
| Dietician |  |  |
| Speech therapist |  |  |
| **Other medical professional -** Please specify which type | | |
|  |  |  |
|  |  |  |
|  |  |  |

**B6. Did you require time off work to attend your appointments?**

☐ Yes ☐ No

If yes, what was the number of days off work? _____________________

What was the estimated wage loss per day off work (post-tax)? $__________________________________________________________

**B7. What is your gross yearly salary?**

☐ <20,000 ☐ 20,000-39,999 ☐ 40,000-59,999

☐ 60,000-79,999 ☐ ≥80,000 ☐ On pension

☐ Don’t know/Don’t want to state this

**B8. Did your partner or close friend or support person require time off work to attend your appointments with you?**

☐ Yes ☐ No

If yes, what was the number of days off work? ______________________

If known, what was the estimated wage loss per day off work (post-tax)?

$__________________________________________________________

**B9. Did you require accommodation when coming for your appointments?**

☐ Yes ☐ No

1. If yes, how much did accommodation cost you per hospital or specialist visit? $_________________________________________________________
2. How many visits required you to pay for accommodation? ☐ 1 ☐ 2 ☐3

☐ 4 ☐ 5 ☐ 6 ☐ 7 ☐ 8 ☐ 9 ☐ 10 ☐ More than 10

1. Were you reimbursed for the travel/accommodation costs? ☐ Yes ☐ No
2. Did this involve the Patient Assistance Transport Scheme (APTS) form?

☐ Yes ☐ No

**B10. Did your partner or closest friend or support person require accommodation when coming for your appointments?**

☐ Yes ☐ No

1. If yes, how much did accommodation cost them per hospital or specialist visit? $_________________________________________________________
2. How many visits required them to pay for accommodation? ☐ 1 ☐ 2 ☐3

☐ 4 ☐ 5 ☐ 6 ☐ 7 ☐ 8 ☐ 9 ☐ 10 ☐ More than 10

1. Were they reimbursed for the travel/accommodation costs?

☐ Yes ☐ No

1. Did this involve the Patient Assistance Transport Scheme (APTS) form?

☐ Yes ☐ No

**B11. Childcare arrangements or informal care duties (looking after elderly relative).** *Here we are interested only in assistance that you needed in addition to any usual childcare or informal care arrangements.*

1. When you were in hospital or at home recovering from the operation, did you usually get someone to look after your child/children or other dependants (if you have any)?

☐ Yes ☐ No ☐ Not applicable (no children or other dependents)

1. If yes, how much do you estimate you paid the caregiver (total)? $________________________________________________________

Or what was the wage loss for the caregiver? $___________________

**B12. Did you need any care from an agency (e.g. District Nurse) or professional care giver?**

☐ Yes ☐ No

1. How many care visits did you require in total? _____________________
2. How much did you estimate each visit cost?

$__________________________________________________________

**B13. How many medicines were prescribed FOR YOU by your doctor during the course of your treatment and how many did you pay for?** *Please include all repeat prescriptions*

1. Number of prescribed medicines: ________________________________
2. Number of repeats used per medication: __________________________
3. How many medications did you pay for including repeats? ____________
4. How much did it cost you each week total? $________________________
5. Did you use a concession card? ☐ Yes ☐ No

**B14. In total how much have you spent on medication bought over-the-counter during the course of your treatment?**

1. Total cost: $ ______________________________________

**B15. During the course of your treatment, how many times (if any) were you admitted to a day unit, a hospital, or to accident and emergency (A&E)? If you were admitted to hospital, please also state the total number of days spent in hospital.**

| **Type of Admission** | **Number of times** | **Total number of days in hospital** | **N/A** |
| --- | --- | --- | --- |
| Day unit |  |  | ☐ |
| Hospital |  |  | ☐ |
| Emergency or A&E or casualty |  |  | ☐ |

**B16. How many ambulances did you require and how much did it cost you per ambulance?**

☐ 1 ☐ 2 ☐3 ☐ 4 ☐ 5 ☐ 6 ☐ 7 ☐ 8 ☐ 9 ☐ 10 ☐ More than 10 ☐ N/A

$_______________________________________________________.

**B17. During the course of your treatment, has someone outside the household helped you with any of the household tasks you would have usually done yourself?**

☐ Yes ☐ No

A) If yes, what was the total cost? $_________________________________

**B18. During the course of your treatment did you need to obtain any aids/appliances or modifications to help you in the home (e.g. kitchenware, bread oven, blender)?**

☐ Yes ☐ No

A) If yes, what was the total cost? $_________________________________

**SECTION D: PERSONAL CHARACTERISTICS**

**D1. What is today’s date?**  ……….…/……………/……..… (day/month/year)

**D2. What is your age (years)?**

☐ 40-49 ☐ 50-59 ☐ 60-69

☐ 70-79 ☐ 80-89

**D3. What is your gender?**

☐ Male ☐ Female ☐ Prefer not to say

**D4. What is your marital status?**

☐ Married/de-facto ☐ Single (never married)

☐ Widowed ☐ Divorced/Separated

**D5. Were you born in Australia?**

☐ No ☐Yes

**D6. Do you identify as an Aboriginal or Torres Strait Islander** **Australian?**

☐ Aboriginal ☐ Torres Strait Islander

☐ Both ☐ Neither

**D7. What is the highest level of education that you completed?**

☐ Primary school ☐ Secondary school ☐ Trade or Diploma

☐ Bachelor’s degree ☐ Postgraduate Degree

**D8. What is the postcode of the suburb/town in which you live?**

_____________________________________________________________________________

**D9. What percentage of total income in your household was taken up by out-of-pocket treatment costs?**

☐ 5% ☐ 10% ☐ 20%

☐ 50% ☐ 80% ☐ 100%

**B10. When were you diagnosed with oesophageal cancer?**

_____________________________________________________________________________

**D11. Do you wish to receive a copy of the summary sheet of the results of this study mailed to you at the conclusion of this study?**

☐ Yes

☐ No

***Thank you for taking time to complete this questionnaire!***

***Please post the completed questionnaire back to us in the enclosed, pre-paid envelope.***

**Supplementary Table S1**. The breakdown of total out-of-pocket costs based on the components across all participants, and by insurance type.

| **Components** | **All participants** | | | **Public** | | | **Private** | | | **P-value (Public vs Private)** |
| --- | --- | --- | --- | --- | --- | --- | --- | --- | --- | --- |
|  | Total cost all (AUD) | % | Median (95%CI) AUD | Public total cost (AUD) | % | Median (95%CI) AUD | Urban total cost (AUD) | % | Median (95%CI) |  |
| Medical | $74,625 | 7.9 | 514(308-890) | $42,825 | 5.8 | 360(220-622) | $31,800 | 15.7 | 1048(664-2292) | 0.0 |
| Travel | $30,813 | 3.3 | 67(35-121) | $17,674 | 2.6 | 60(31-123) | $13,139 | 6.5 | 99(17-334) | 0.8 |
| Accommodation | $4,100 | 0.4 | 0 (0-0) | $1,700 | 0.2 | 0 (0-0) | $2,400 | 1.2 | 0 (0-0) | 0.8 |
| Wage loss | $611,056 | 64.7 | 0 (0-0) | $457,415 | 61.7 | 0 (0-0) | $153,641 | 76.0 | 0 (0-0) | 0.5 |
| Carer cost | $223,598 | 23.7 | 0 (0-0) | $222,318 | 30.0 | 0 (0-0) | $1,280 | 0.6 | 0 (0-0) | 0.1 |
